# Ferroptosis Modulation: Potential Therapeutic Target for Glioblastoma Treatment

**DOI:** 10.3390/ijms23136879

**Published:** 2022-06-21

**Authors:** Izadora de Souza, Maria Carolina Clares Ramalho, Camila Banca Guedes, Isabeli Yumi Araújo Osawa, Linda Karolynne Seregni Monteiro, Luciana Rodrigues Gomes, Clarissa Ribeiro Reily Rocha

**Affiliations:** 1 Department of Clinical and Experimental Oncology, Federal University of Sao Paulo (UNIFESP), Sao Paulo 04037-003, Brazil; izadora.souza@unifesp.br (I.d.S.); camila.guedes@unifesp.br (C.B.G.); linda.seregni@unifesp.br (L.K.S.M.); 2 Laboratory of Cell Cycle, Center of Toxins, Immune Response and Cell Signaling (CeTICS), Butantan Institute, Sao Paulo 05503-001, Brazil; clares.carolina@unifesp.br (M.C.C.R.); isabeli.osawa@unifesp.br (I.Y.A.O.); luciana.gomes@butantan.gov.br (L.R.G.)

**Keywords:** ferroptosis, glioma treatment, cell death

## Abstract

Glioblastoma multiforme is a lethal disease and represents the most common and severe type of glioma. Drug resistance and the evasion of cell death are the main characteristics of its malignancy, leading to a high percentage of disease recurrence and the patients’ low survival rate. Exploiting the modulation of cell death mechanisms could be an important strategy to prevent tumor development and reverse the high mortality and morbidity rates in glioblastoma patients. Ferroptosis is a recently described type of cell death, which is characterized by iron accumulation, high levels of polyunsaturated fatty acid (PUFA)-containing phospholipids, and deficiency in lipid peroxidation repair. Several studies have demonstrated that ferroptosis has a potential role in cancer treatment and could be a promising approach for glioblastoma patients. Thus, here, we present an overview of the mechanisms of the iron-dependent cell death and summarize the current findings of ferroptosis modulation on glioblastoma including its non-canonical pathway. Moreover, we focused on new ferroptosis-inducing compounds for glioma treatment, and we highlight the key ferroptosis-related genes to glioma prognosis, which could be further explored. Thereby, understanding how to trigger ferroptosis in glioblastoma may provide promising pharmacological targets and indicate new therapeutic approaches to increase the survival of glioblastoma patients.

## 1. Introduction

Glioblastomas (GBM, WHO IV) are the most aggressive type of glioma due to their peculiar characteristics such as cellular heterogeneity, high proliferation rate, diffuse infiltration capacity, and more importantly, high resistance to chemotherapeutic drugs [1]. Therefore, the standard treatment with temozolomide (TMZ) has low effectiveness, leading to a poor prognosis with a median overall survival of not more than 15 months, and a five-year survival rate of only 5% [2]. Indeed, it has been a challenge to sensitize this type of tumor due to its malignant attributes, and therefore, strategies involving the induction of cell death have been widely studied. Undoubtedly, one of the most studied cell death targets for tumor therapy is apoptosis, however, glioma cells have developed several mechanisms of resistance toward this type of cell death [3,4]. Thus, focusing on studying different types of cell death that still have not yet been completely characterized is a relevant tool in glioma treatment strategies.

At its most basic, cell death is an important biological mechanism for physiological homeostasis, which may occur in response to stress conditions [5]. Upon oxidative stress, a specific group of effector molecules is activated and triggers a cell death signaling pathway in a programmed manner to preserve the organism’s integrity. Thus, cell death is extremely important to eliminate damaged cells, and to regulate the state of danger signaling. According to Nomenclature Committee on Cell Death, there are several types of regulated cell death including apoptosis, necroptosis, autophagy, and ferroptosis. Regulatory failure in these different types of cell death can lead to human disorders [6]. Thus, the research on the molecular mechanisms of cell death is an important strategy to better understand the pathologies and promote new treatment options for aggressive diseases such as glioblastoma. 

**Figure 1:**
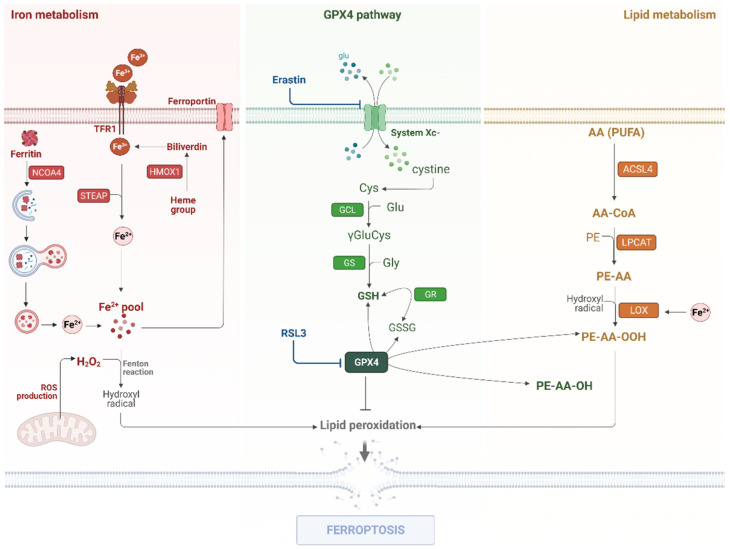
**The molecular mechanisms of the ferroptosis pathway.** Ferroptosis is triggered by three main regulatory pathways: iron metabolism, the GPX4 pathway, and lipid metabolism. In the iron metabolism, Fe^3+^ (ferric iron) is transported into the cell by TfR1 (Transferrin receptor) or obtained through the conversion of the heme group to biliverdin by HMOX1 activity. Then, Fe^3+^ is reduced to Fe^2+^ (ferrous iron) by STEAP3. Ferritin degradation by NCOA4 via autophagy also contributes to the labile iron pool. Once in the cytosol, Fe^2+^ can react with ROS and it generates the hydroxyl radical, promoting PUFA oxidation. The GPX4 pathway is responsible for controlling the lipoperoxidation levels through the reduction of lipid peroxides (PE-AA-OOH) to lipid alcohol (PE-AA-OH), thus GPX4 blockage by RSL3 induces ferroptosis. GPX4 utilizes GSH as a cofactor, therefore the GSH synthesis pathway is directly related to GPX4 activity, and xCT system blockage by Erastin leads to ferroptotic cell death. In the lipid metabolism, AA (as well as other PUFAs) are metabolized by ACSL4 and esterified by LPCAT3. Then, LOXs oxidize PUFAs using Fe^2+^ as a cofactor, which produces lipid peroxides. Created with BioRender.

Studies have revealed an emergent type of regulated cell death identified as ferroptosis (Figure 1), which may play an important role in cancer treatment [7]. Ferroptosis has been initially described in a study by Dixon et al., in which the researchers used several lethal molecular compounds to kill *RAS*-mutated cancer cell lines [8]. As a consequence, two main compounds, namely, Erastin and RSL3, were identified. They promote non-apoptotic regulated cell death, and the process was termed ferroptosis by the authors. In that study, the authors distinguished this new type of cell death both morphologically and biochemically from other regulated cell deaths [8].

Over the past years, a large body of experimental evidence has become available regarding ferroptosis characterization. Thus, it is now clear that the main mechanisms to trigger ferroptosis involve increased intracellular labile iron, oxidized polyunsaturated fatty acids (PUFAs) associated with phospholipids, and deficient repair of lipid peroxides by GPX4 (glutathione peroxidase 4). These pathways contribute in the promotion of lethal lipid peroxidation, which in turn leads to membrane cellular disruption, and consequently, cell death [9].

Lipid peroxidation is essential for triggering ferroptosis and it occurs in three phases: initiation, propagation, and termination [10]. In the initiation phase, reactive oxygen species (ROS) such as hydroxyl radicals (OH) and hydroperoxyl radicals (OOH), reactive nitrogen species (RNS), and reactive lipid species in elevated levels react with a hydrogen atom from an allylic carbon of membrane PUFAs, resulting in a lipid radical. The key initiators of lipid peroxidation are ROS and iron ferrous (Fe^2+^), which reacts to form a hydroxyl radical by the Fenton reaction [i.e., the interaction of ferrous iron (Fe^2+^) with hydrogen peroxide (H_2_O_2_)] [11]. During the propagation phase, the lipid radical is susceptible to react with oxygen, forming a peroxyl radical (LOO), which can interact with another allylic carbon from PUFAs to generate a new lipid radical and lipid peroxide (LOOH). The termination phase occurs when two or more lipid peroxyl radicals reach elevated concentrations to react with antioxidants such as GSH (glutathione) and GPX4 to form non-radical products, leading to the termination of this process in physiological conditions and, consequently, inhibiting ferroptotic cell death [11]. If the termination phase is interrupted by ferroptosis inducers, lipid peroxides can be degraded to malondialdehyde (MDA) and 4-hydroxy-2-nonenal (4-HNE). These molecules are responsible for signaling events of cell death initiation by promoting toxicity in key proteins [12].

Since GPX4 is the key enzyme involved in the lipid peroxidation control, its inhibition or inactivation can trigger the ferroptosis pathway. Indeed, ferroptosis inducers can promote ferroptosis through the depletion of GSH and, consequently, GPX4 inactivation, or by direct GPX4 inhibition, indicating GPX4 as a pivotal regulator of ferroptosis across a wide range of cell lines [13]. GPX4-knockout mice were susceptible to ferroptosis, indicating the pathological relevance of this cell death [14]. In this sense, compounds that inhibit lipid peroxidation such as ferrostatin-1 and liproxstatin-1 can suppress ferroptosis in GPX4-deficient cells [14]. In physiological conditions, lipophilic antioxidants such as vitamin E and coenzyme Q10 are important detoxifying agents [15].

Currently, the utilization of ferroptosis-inducing drugs in cancer have been widely studied as a promising therapeutic approach to eradicate resistant cancer cells [16]. Notably, glioblastoma cells have elevated levels of intracellular ROS and higher iron metabolic demand in comparison to healthy tissues, which promotes more susceptibility to ferroptosis induction [17,18]. Therefore, there are some already known strategies to induce ferroptosis through canonic pathways in cancer cells by increasing the intracellular iron levels [19] and targeting GPX4 through several mechanisms [9]. In addition to the direct or indirect blockage of GPX4 by ferroptosis-inducing compounds, the inhibition of GPX4 expression by modification in the mevalonate pathway also promotes ferroptosis in cancer cells [15]. 

Likewise, the inhibition of GSH synthesis by buthionine sulfoximine (BSO) or depletion of extracellular cystine by cyst(e)inase also promotes GSH depletion and ROS accumulation, which can potentiate the sensitivity of cancer cells to ferroptosis inducers and suppress tumor growth, since GPX4 requires GSH as a cofactor [20,21]. Additionally, targeting non-canonic pathways has demonstrated significant results in ferroptosis modulation, as mentioned below in detail. 

Investigating the molecular mechanisms of ferroptosis provides a better understanding of tumor development and, consequently, leads to the identification of new potential targets. Indeed, ferroptosis has been increasingly studied as an attractive way to prevent the proliferation and invasion of resistant tumors such as glioblastoma [16]. Furthermore, it has been demonstrated that glioma cells can effectively escape from the ferroptosis process, increasing their aggressiveness and chemoresistance [16]. Therefore, increasing ferroptosis activity resulted in decreased tumor proliferation in glioblastoma cells, which indicates a potential therapeutic target in GBM to improve the current non-effective treatments [22,23,24,25]. Thus, in this comprehensive review, we explore the current findings regarding the ferroptosis pathway, which may offer new insights for glioma treatment.

## 2. Methodology

In this review, we focused on demonstrating the current scenario of research regarding the role of ferroptosis in glioblastoma. For this purpose, we used the PubMed database to search the biomedical literature regarding ferroptosis pathways in glioblastoma. Briefly, we searched the keywords ‘GBM or Glioblastoma and Ferroptosis’ and ‘Glioma and Ferroptosis’. Then, the most relevant papers published from 2013 to May 2022 were selected. Terms related to the hallmarks of ferroptosis such as ‘GPX4 pathway’, ‘system xc’, ‘NRF2′, ‘Iron metabolism’, ‘Fenton reaction’, ‘ferritin’, ‘ferritinophagy’, and ‘lipid metabolism’ were also searched. The terms ‘FSP1′, ‘BH4′, ‘DHODH’, ‘CoQH2′, and ‘Metabolic Regulation of Ferroptosis’ were searched to mention the secondary pathways associated with ferroptosis, and the term ‘ferroptosis and neurodegeneration’ to discuss the side-effects associated with drugs targeting ferroptosis. After that, the articles regarding the bioinformatics analysis of ferroptosis biological markers were separated from those regarding the potential ferroptosis-inducing compounds for the tables’ construction. All figures were created with BioRender. 

## 3. Ferroptosis Modulation on Glioma

### 3.1. Iron Metabolism

Iron is commonly associated with its role in oxygen transport in the blood, however, this element is involved in many other biological processes including nucleic acid repair, DNA synthesis, cell growth, and cell death [26]. The level of iron is maintained by the action of several regulatory proteins. Initially, for intestinal absorption to occur, dietary iron is reduced to Fe^2+^ (ferrous iron) by ferric reductase duodenal cytochrome B (DCYTB) activity. After its reduction, Fe^2+^ is absorbed by a divalent metal transporter (DMT1), located in enterocytes, and it can be redirected to three distinct purposes: (1) the storage in ferritin protein; (2) the execution of biological processes in the cells; or (3) the release in the blood circulation [26].

Iron is exported from enterocytes by ferroportin (FPN1) and oxidized to Fe^3+^ (ferric iron) by the Hephaestin protein (Hp), enabling its binding to the glycoprotein transferrin (Tf). In cells where Transferrin receptors (TfR1) are present such as erythroblast precursors, Tf is internalized. The iron is released in the endosome and, by the action of metalloreductase six-transmembrane epithelial antigen of the prostate 3 (STEAP3), it is reduced to Fe^2+^, allowing its cytosol exportation by DMT1. When it is in cytosol, ferrous iron can be used in hemoglobin heme biosynthesis or it can be stored in ferritin, depending on the cellular needs [26]. To maintain adequate iron levels, the ferritin-stored iron can be released by ferritinophagy, an autophagic process mediated by NCOA4 proteins [27].

Despite its participation in several essential biological processes, excess iron can be harmful to cells. Indeed, iron can promote the oxidation of biomolecules, generating reactive oxygen species (ROS). Furthermore, iron is capable of reacting with ROS such as hydrogen peroxide (H_2_O_2_) and oxygen (O_2_), promoting the formation of hydroxyl radicals (OH) and anion superoxide (O_2_^−^) by the Fenton chain reaction [28]. This increase in hydroxyl radicals, resulting from iron accumulation, promotes PUFA oxidation, which is a well-established ferroptosis hallmark [9].

An expanded labile iron pool (LIP) has been considered as a notable characteristic that distinguishes cancer cells from normal cells, since all cancer cells are more dependent on iron than normal tissues to support the intense energetic demand that comes with indefinite proliferation. Due to this, ferroaddiction in cancer has been explored in LIP-targeted therapies that induce oxidative stress to trigger ferroptosis. This approach requires biomarkers to point out those tumors with the most elevated LIP, and thus most likely to respond to LIP-targeted therapies [29,30]. In this sense, a radiotracer was developed, named 18F-TRX, to assess LIP in situ with PET, and showed that among other types of cancer, glioma harbors a wide range of LIP concentrations. U251 cells had the highest 18F-TRX uptake and were highly sensitive to treatment with TRX-CBI—a LIP-activated prodrug—suggesting a role for LIP-targeted therapies in the treatment of glioma [30].

It is known that GBM cells accumulate iron by altering the expression levels of many proteins and enzymes related to iron metabolism, promoting physiological processes such as tumor initiation, progression, and metastasis [29]. One way to increase the iron content in cancer cells is through TMZ treatment. According to a recently published study, TMZ, which is a standard chemotherapeutic drug utilized in glioblastoma treatment, drives ferroptosis by upregulating DMT1, a transporter related to iron metabolism. Thus, DMT1 could be a crucial target in GBM [31]. In this context, Zhang and collaborators revealed that the overexpression of the Coatomer protein complex subunit zeta 1 (*COPZ1*), a component of the coatomer protein complex I, was associated with increasing tumor grade and poor prognosis in glioma patients [32]. The referred study demonstrated that *COPZ1* knockdown induced ferritinophagy, and ultimately, activated cell death via ferroptosis in the U87MG, U251, and P3#GBM cultured cell lines. Additionally, the deficiency of *COPZ1* led to increased levels of the proteins NCOA4, autophagy flux marker LC3B-II, and ATG7 (autophagy-related 7), resulting in the degradation of the intracellular iron storage protein ferritin through autophagy, and consequently, augmented levels of ferrous iron. Therefore, iron accumulation generated high levels of intracellular H_2_O_2_ and superoxide, triggering the Fenton reaction and leading to lipid peroxidation [32].

In the same way, *STEAP2* and *STEAP3*, identified as potential prognostic-related genes in GBM, were found to be downregulated and upregulated, respectively, in these tumors [33]. As members of the six-transmembrane epithelial antigen of prostate (STEAP) family, these proteins play a significant role in maintaining iron homeostasis, reducing ferric iron to ferrous iron to increase cellular iron uptake [33,34]. Considering the link between iron metabolism and ferroptosis, the Pearson’s correlation analysis indicated that *STEAP2* and *STEAP3* were correlated with genes involved in ferroptosis [35] such as *ACSL4, ALOX5, CBS, FANCD2, GCLM, HMGCR, HSPB1, NFE2L2, PTGS2*, and *SAT1* [33]. Additionally, via Gene Ontology (GO) enrichment analysis, both genes were associated with immune regulation and cell cycle transition in the initiation and progression of GBM, but the mechanisms involving this association require further investigation [33].

According to Zhang and co-workers, ferroptosis has a therapeutic effect in glioblastoma, and synergistic effects when combined with chemotherapeutic agents [36]. This report found that gallic acid (GA), a natural compound extracted from gallnut, can complex with Fe^2+^ to form nanoparticles (GA/Fe^2+^ nanoparticles, GFNPs), which can lead to GBM cell ferroptosis by promoting the Fenton reaction. In addition, GA can effectively reduce Fe^3+^ to Fe^2+^, inducing the Fenton reaction even further [36]. Based on the highly stable Fenton catalytic activity of GFNPs, a biocompatible nanodrug known as cRGD/Pt + DOX@GFNPs (RPDGs) was designed as a potential anticancer strategy, combining the action of two types of cell death: apoptosis and ferroptosis. The nanoparticle consists of the cyclic Arg-Gly-Asp peptide (cRGD) that can mediate the nanodrug endocytosis by binding to α_v_β_3_ integrin, which is highly expressed on the surfaces of the tumor cells and neovascular endothelial cells; Pt (IV) which is reduced to Pt (II), causing the depletion of GSH and significant increase in the intracellular ROS levels in the process; doxorubicin (DOX), a broad-spectrum chemotherapeutic drug; and the GFNPs. Both cRGD and Pt (IV) were functionalized with DSPE-PEG (2000). Other than elevating the levels of Fe^2+^ and triggering the Fenton reaction, the RPDGs increased the intracellular ROS levels and generated lipid peroxidation, inducing significant ferroptosis [36].

Furthermore, a study revealed that upon endoplasmic reticulum (ER) stress induced by brucine, activating transcription factor 3 (*ATF3*) was upregulated and translocated to the nucleus of glioma cells. Then, *ATF3* contributed to the intracellular accumulation of H_2_O_2_ by upregulating *NOX4* and *SOD1*, and downregulating *xCT* and catalase. As a member of the NADPH oxidase family, *NOX4* is responsible for the conversion of superoxide to H_2_O_2_ in the presence of cytoplasmic *SOD1* and it was found to be overexpressed in human gliomas. In the end, high levels of H_2_O_2_ led to brucine-induced ferroptosis in glioma cells through the upregulation of *TFR* and the consequent iron overload, which also caused lipid peroxidation [37].

### 3.2. Lipid Metabolism

Since PUFAs play a pivotal role in ferroptosis, lipid metabolism is directly related to the ferroptosis pathway. This type of fatty acid is much more susceptible to oxidation than saturated fatty acids or monounsaturated fatty acids due to the bis-allylic hydrogen atoms present within its molecular structure [10]. The polyunsaturated fatty acid chain can be incorporated into phospholipids of the cellular membrane, becoming susceptible to oxidation. It has been established that this incorporation is mediated by *ACSL4* and *LPCAT3* and the deletion of these genes can prevent ferroptosis [38]. ACSL4 catalyzes arachidonic acid (AA) and adrenic acid (AdA) reactions and produces acyl Co-A. Then, *LPCAT3* is responsible for esterifying these fatty acids into phosphatidylethanolamines (AA-PE and AdA-PE), which are oxidized by *ALOX15*, generating lipid hydroperoxides [39,40,41].

ACSL4 knockout cells can lose their sensitivity to ferroptosis upon supplementation with exogenous AA and AdA [40]. Recently, it has been demonstrated that the microRNA-670-3p can suppress ferroptosis through *ACSL4* inhibition in human glioblastoma cell lines U87MG and A172, therefore miR-670-3p inhibitors increased the sensitivity to temozolomide treatment [42]. Additionally, *ACSL4* overexpression has promoted high levels of lipid peroxides and cell viability reduction in glioma cells, suggesting a central regulatory role of this gene in ferroptosis modulation on brain tumors, which may serve as a potential target [43]. Important roles have also been assigned to lipoxygenase enzymes (LOXs) in cancer development in different pathways. In ferroptosis, these enzymes oxidized fatty acids, triggering lipid peroxide formation using Fe^2+^ as a cofactor [44]. *ALOXE3* inhibition by miR-18a activity promotes the resistance to ferroptosis in GBM cells, which increases cell survival and migration [45]. 

Other lipid-related genes have also promoted ferroptosis modulation. For instance, *CYP2E1* activity produces acetaldehyde and ROS, increasing lipid peroxidation and promoting ferroptosis. Glioma patients with low levels of *CYP2E1* have a poor prognosis since the downregulation of this gene affects lipid metabolism and prevents ferroptosis in tumor tissues, leading to glioma progression [46]. Additionally, *MDM2* and *MDMX* promote ferroptosis in a p53-independent manner by modulating lipid activity through PPARα regulation and by inhibiting the activity of lipophilic antioxidants via *FSP1* protein regulation [47].

### 3.3. The GPX4 Pathway

The defect in the repair system that removes lipid hydroperoxides of PUFA-PLs is another important ferroptosis hallmark. Glutathione peroxidase 4 (GPX4), one enzyme member of the GPX family, stands out due to its phospholipid hydroperoxidase activity, protecting the cells from oxidative damage caused by ROS and thus maintaining cellular lipid homeostasis [9]. Currently, GPX4 is an important regulator of ferroptosis, since it converts lipid hydroperoxides to lipid alcohol, preventing these molecules from triggering the process of lipid peroxidation [48]. In order to eliminate lipid hydroperoxides, GPX4 reduces GSH to oxidized gluthatione (GSSG), thus GSH-depleted cells usually die on account of ferroptosis [49]. GSH is an essential cellular antioxidant, acting in the reduction of oxygen radicals and maintaining the cell’s redox balance. The synthesis of GSH occurs in two steps, catalyzed by γ-glutamylcysteine ligase (GCL) and GSH synthetase. GCL is formed by two subunits, one catalytic (GCLC) and one reductive (GCLM) [50]. Low GSH activity can increase the oxidative stress and lead to cell death [51].

Recent studies have shown that some neuroprotectors such as tert-butylhydroquinone (tBHQ), 15-deoxy-prostaglandin J2, curcumin, and melatonin are involved in the increased production of GCLC, a precursor of glutathione. These molecules protect cells from oxidative stress, which is one of the features of ferroptosis induction [51]. Therefore, compounds that influence GSH synthesis are associated with cell resistance, mediating ferroptosis. Recently, it has also been demonstrated that gastrodin can diminish lipid peroxidation and prevent ferroptosis in glioma [52], indicating new targets to ferroptosis modulation.

The increase in intracellular Fe^2+^ associated with GSH depletion results in an increase in ROS and lipid peroxidation, which are crucial factors in the induction of ferroptosis [53]. Higher GPX4 expression was observed in human glioma cells (U251 and U87) compared to normal glial cells, suggesting that ferroptosis sensitivity is reduced in this type of tumor [54]. Assuming that the inhibition of GPX4 leads to the accumulation of lipid peroxides, which in turn cause damage to cellular lipid membranes, inducing ferroptosis, the modulation of GPX4 is a potential therapeutic strategy against many cancer types including glioblastoma. Therefore, the GPX4 blockage or inhibition of GSH synthesis is a relevant mechanism to be studied [48,55].

Cysteine depletion is another factor that results in lipid peroxides. Cystine arrives in the cell through the system xCT, where it will be used as a substrate to produce reduced GSH. The system xCT is an antiporter of glutamate-cysteine, through which the cell internalizes cystine in exchange for glutamate. In turn, cystine is reduced to cysteine, which is essential in the production of GSH. Due to its important participation in the antioxidant defense, xCT has become a promising therapeutic target in gliomas [23]. Thus, one of the strategies for this is the blockage of the cystine capture, which presents an indirect way to inhibit GPX4 through the inhibition of xCT [56]. The system xCT—or “cystine/glutamate antiporter system xc-”—is formed by the regulatory subunit *SLC3A2* (also known as a 4F2 heavy chain, 4F2hc, or CD98) *SLC7A11* gene that encode the transport subunit xCT [56,57,58].

Recently, pharmacological ferroptosis inducers for glioblastoma treatment that operate through the blockage of the system xCT such as Erastin [59], sulfasalazine [60], or sorafenib [61] have been explored [62]. Interestingly, such ferroptosis inducers could potentiate temozolomide toxicity [22,63]. In addition, some studies have shown that *xCT* expression could be modulated by *ATF4* [64]. The activating transcription factor 4 (*ATF4*) is related to cellular homeostasis, protecting the cell against oxidative stress. Studies have demonstrated that its high expression is associated with glioma malignancy, increasing angiogenesis and tumor proliferation [64]. The glutamate antiporter (xCT) is highly expressed when *ATF4* is activated, and the higher level of *xCT* induces greater resistance to TMZ [65]. It was observed that the cells became more sensitive to the ferroptosis inducers, sorafenib, Erastin, and RSL3, with the knockdown of *ATF4* [64]. *ATF4* is described as a chemoresistance gene in gliomas, because its high expression promotes glioma resistance to TMZ [65]. Recent studies have demonstrated that cystine deprivation induces ferroptosis in T98G and A172 cells [66]. Curiously, cell lines treated with BSO escaped from cell death when they received inducers to increase the level of intracellular iron such as ferrous ammonium sulfate (FAS) or hemin. Thus, this suggests that ferroptosis-induced cystine deprivation requires not only a decrease in GSH, but also intracellular iron accumulation [66].

Another factor that can be considered as one of the keys to ferroptosis regulation is the transcription factor *NRF2* due to its role in controlling the expression of intracellular redox-balancing proteins including GPX4 and *SLC7A11* [67]. *NRF2* also represents a potent mechanism of resistance in glioma [68,69]. Of note, *NRF2* overexpression promoted higher proliferation, oncogenic transformation, and ferroptosis resistance in glioma cells through *xCT* upregulation [23]. However, *NRF2* could play a contrasting role in ferroptosis by *ABCC1*/MRP1 upregulation, a pro-ferroptotic target that could induce ferroptosis by GSH depletion, or by the HMOX1 regulation, promoting high iron levels [70,71] [unpublished observations]. Altogether, these findings demonstrate that Nrf2 is an interesting pathway to be further explored in ferroptosis modulation in glioma.

Different studies have associated the compound RSL3 with the inhibition of the GPX4 activity. Recent data associated RSL3 activity with the activation of the NF-kB pathway and the depletion of GPX4, which induced lipid peroxidation in glioblastoma cells, reducing proliferation [72]. Xuanzhong Wang and collaborators demonstrated that this compound decreases the cellular viability of glioblastoma in vitro and in vivo. Additionally, RSL3 promotes cell death in a dose-dependent manner in the U373, U87, and U251 lines [72]. Similarly, other results have demonstrated that the U87MG cell line was sensitive to ferroptosis induced by the direct inhibition of GPX4 by RSL3 treatment [55], and murine glioma GL261 cells died through ferroptosis after RSL3 stimulation [73].

#### The GPX4-Independent Pathways

Although GPX4 has played a central role in ferroptosis suppression, the sensitivity toward GPX4 inhibitors varies across a wide range of cancer cell lines, which suggests that other pathways could be related to lipid peroxidation control and, consequently, to prevent ferroptosis in cancer. In this regard, recently, it has been described as an independent-GPX4 pathway of lipid peroxidation prevention via ferroptosis suppressor protein 1 (*FSP1*) activity [74,75]. Studies have identified that *FSP1* confers resistance against ferroptosis upon treatment with GPX4 or xc-system inhibitors in vitro and in vivo, and its depletion can elevate lipid peroxide levels in cancer cells [74]. FSP1 works as an oxidoreductase reducing ubiquinone (Coenzyme Q10) to its antioxidant form ubiquinol (CoQH2), which acts as a lipophilic radical scavenger that limits the lipid peroxide accumulation in the absence of GPX4, or complementary to GPX4 activity [75]. Interestingly, pharmacological inhibitors of FSP1 such as iFSP1 synergizes with GPX4 modulation to trigger ferroptosis in *FSP1* overexpressed cells [76,77], and iFSP1 can sensitize several cancer cells to ferroptosis [78]. Similarly, dihydroorotate dehydrogenase (DHODH) has also demonstrated an anti-ferroptotic role by regulating ubiquinol activity, inhibiting lipid peroxidation and suppressing ferroptosis induction [79].

The GTP cyclohydrolase-1 (GCH1)-tetrahydrobiopterin (BH4) pathway has also lately been associated with ferroptosis suppression in a genome library CRISPR/Cas9 screening study [80]. BH4 is an antioxidant protein that limits oxidative damage during ferroptosis [81] and its biosynthesis is an essential metabolic pathway upon GPX4 inhibition [82]. GCH1 is crucial for BH4 synthesis, therefore, *GCH1* overexpression strongly inhibits ferroptosis in a GPX4-independent manner by inhibiting PUFAs-PL oxidation [80]. Likewise, dihydrofolate reductase (DHFR) is associated with ferroptosis prevention since this enzyme contributes to the regeneration of BH4 and supports its function against ferroptosis [82]. 

Altogether, these findings indicate that the inhibition of these secondary pathways could represent a promising therapeutic target by decreasing the resistance to ferroptosis and also by potentiating the treatment effect when administrated with ferroptosis-inducing compounds [75]. Nevertheless, up to now, the evidence regarding how these GPX4-independent pathways act in glioblastoma and how they can be modulated to improve the current treatments remains unclear, indicating the need for further investigation in this field.

## 4. Non-Canonical Pathways

### 4.1. LncRNAs, CircRNAs, and miRNAs

Currently, many authors have established ferroptosis-related lncRNA signatures to predict the prognosis of glioma patients, enabling a more individualized treatment based on the risk score. This approach is fundamental for the development of therapies that target these lncRNAs. In one particular study, 14 long non-coding RNAs (lncRNAs) were found to develop a signature capable of estimating tumor progression in glioma patients. This panel includes lncRNAs, most of which are related to cell migration and invasion, proliferation, and tumor progression [83]. In another study, it was also demonstrated that a ferroptosis-related lncRNA signature could predict an immune landscape and radiotherapy response in all types of glioma patients [84]. According to transcriptomic data, 15 lncRNAs were selected to compose a signature that predicts survival outcomes. The high-risk patient’s group had upregulation of risky lncRNAs, while the low-risk patients demonstrated upregulation of protective lncRNAs and also showed a better response to radiotherapy and a different immune landscape [84].

The lncRNAs’ differential expression may also be related to chemoresistance and the biological behavior of cancer cells. Overexpression of TMEM161B-AS1 in U87 and U251 cells is associated with cell proliferation, migration, and invasion. TMEM161B-AS1 regulates the expression of FANCD2 (Fanconi anemia complementation group D2) and CD44, both ferroptosis-related genes, by sponging hsa-miR-27a-3p, promoting a malignant biological behavior and temozolomide (TMZ) resistance [85].

Circular RNAs (circRNAs) are also biomarkers in glioma for presenting an essential function in tumorigenesis regulation. Studies have shown an upregulation of circ-TTBK2 in glioma cells and tissues, accompanied by an increase in ITGB8 (integrin subunit beta 8) and downregulation of miR-761. Knockdown of circ-TTBK2 promotes a decrease in cell proliferation and invasion and the induction of ferroptosis. Circ-TTBK2 is a sponge for miR-761 to modulate ITGB8, explaining the network between these genes [86]. The circular RNA circCDK14 was associated with lower ferroptosis sensitivity, tumoral progression, and malignant behavior in glioma. CircCDK14 promoted the upregulation of PDGFRA, an oncogenic gene, via sponging of miR-3938 [87].

Human glioblastoma tissue presented an increase in the levels of miR-670-3p, but upon ferroptotic stimulation, these levels decreased. This microRNA suppressed ferroptosis by targeting ACSL4, a pro-ferroptotic enzyme [40]. It was demonstrated that the use of miR-670-3p inhibitors promoted the suppression of cell growth and an increase in chemosensitivity to TMZ [42]. In this way, the establishment of a ferroptosis-related lncRNA gene signature to predict the prognosis of glioma patients [84,85,86] as well as the modulation of some circRNAs [86,87] and microRNAs [42] are promising strategies that deserve to be better explored as alternatives to the patients’ resistance to standard therapies.

### 4.2. Autophagy

Autophagy is a lysosome-dependent degradation mechanism essential in maintaining cellular homeostasis. This pathway is activated upon nutrient deprivation, oxidative stress, and DNA damage, for instance, and plays an important role in cellular survival, removing misfolded proteins or damaged macromolecules, organelles, and pathogens. Ferritinophagy is a type of NCOA4 mediated autophagy, which promotes the degradation of the iron storage protein ferritin, increasing the intracellular iron levels [88]. Ferritinophagy has been described as playing a role in cystine deprivation-induced cell death once the inhibition of GSH synthesis is not sufficient to induce ferroptosis in glioblastoma cells [89].

Recently, it was demonstrated that RSL3 and Erastin causes ferroptosis in an autophagy-dependent manner [55,90]. Likewise, novel ferroptotic inducers such as polyphenol amentoflavone (AF) require autophagy activity in order to induce cell death, which was prevented by Ferrostatin-1 [91]. Similarly, the 35G8 induced cell death in U87MG glioblastoma cells was attributed to autophagy and ferroptosis. 35G8 was reported as a nanomolar potent inhibitor of protein disulfide isomerase (PDI), which is an endoplasmic reticulum (ER) oxidoreductase of the thioredoxin superfamily that assists protein folding in the ER and is overexpressed in glioblastoma [63]. 

Furthermore, some studies have established a gene signature that enables the prognosis prediction of glioma patients based on an autophagy-ferroptosis gene profile. So far, 23 autophagy-ferroptosis-related genes including *ATG7, ATG5, LAMP2A*, and *BECN1* have been validated to analyze the prognosis of GBM isocitrate dehydrogenase (IDH) mutated and wild-type as well as oligodendroglioma II/anaplastic oligodendrocytoma III [92]. Another study focused on elucidating the tumor immune escape of patients with glioma based on the autophagy-dependent ferroptosis-related gene (AD-FRG) signature [93]. The establishment of a gene signature is a promising strategy to enable the determination of therapeutic targets and improve the treatment effectiveness in glioma.

## 5. Targeting Ferroptosis for Glioblastoma Treatment and Prognosis

**Figure 2:**
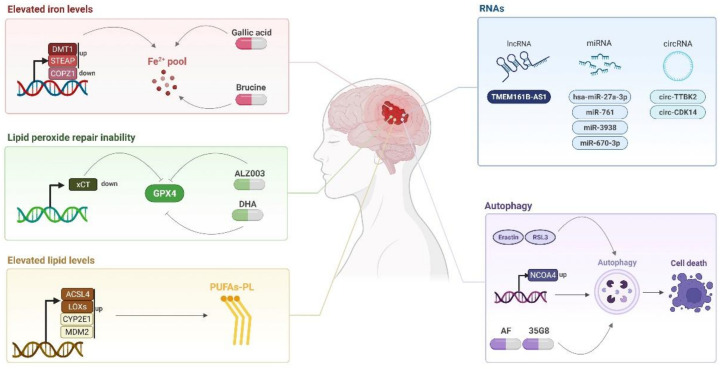
**The schematic view of new potential targets for glioblastoma treatment by ferroptosis modulation.** The red box indicates the interesting new mechanisms for iron accumulation by the upregulation of *DMT1* and *STEAP* genes, or by the downregulation of *COPZ1*. Additionally, the administration of gallic acid and brucine could induce a labile iron pool, promoting ferroptotic cell death in glioblastoma cells. Targeting GPX4 inhibition is also an interesting approach for glioblastoma treatment; this effect could be triggered by *xCT* downregulation, or by AZL003 and dihydroartemisinin (DHA) administration, as demonstrated in the green box. The yellow box shows that the PUFAs accumulation could be induced by the upregulation of several genes of lipid metabolism such as *ACSL4* and *LOXs*, or by *CYP2E1* and *MDM2* upregulation. In the blue box is detailed that the regulation of lncRNA, circRNAs, and miRNAs could also be an interesting target for glioblastoma treatment. Finally, in the purple box, ferroptosis-inducers such as Erastin and RSL3, *NCOA4* regulation, polyphenol amentoflavone (AF), and 35G8 administration promote autophagy induction, leading to ferroptotic cell death in glioma cells. Abbreviations: up: upregulation; down: downregulation. Created with BioRender.

Currently, the GBM treatment consists of surgery for the maximum removal of the tumor, followed by radiotherapy and chemotherapeutic treatment with TMZ, which increases the patient prognosis by approximately 2 months [94]. However, treatment remains inefficient mainly due to drug resistance. Thus, a wide body of research has aimed to target cell death mechanisms, especially apoptosis [95]. However, cells end up developing mechanisms of resistance to this type of cell death. In this sense, ferroptosis modulation becomes an alternative in the treatment of glioblastoma as detailed below and is schematically shown in Figure 2. Recent studies have shown that the increase in ferroptosis in glioblastoma cells was directly correlated with the reduction in tumor growth, providing better outcomes [96]. Likewise, the use of biomarker genes for a better prognosis is also an interesting approach for glioma patients. Several studies have found gene signatures related to ferroptosis to establish a risk assessment of glioblastoma and predict the prognosis [90,97].

### 5.1. Ferroptosis-Inducing Compounds

Compounds capable of inducing ferroptosis can direct new treatments for glioblastoma such as a brucine and cRGD/Pt + DOX@GFNPs nanoformulation, which promotes ferroptosis mediated by the iron pathway [36,37] as well as AF and 35G8, which induced ferroptosis in a autophagy dependent manner [63,91], above-as mentioned. In this sense, next, we present other promising therapeutic compounds for glioblastoma treatment that could reverse chemoresistance through ferroptosis modulation. 

The compound ALZ003 (a curcumin analog), is a negative regulator of GPX4, which promotes a reduction in the GSH/GSSG levels and therefore generates the accumulation of lipid peroxidation and high ROS levels, leading to ferroptosis in the U87MG cell line [98]. Interestingly, natural plant extracts are prospective therapies in many types of cancer including glioblastoma. Artemisinin is an active ingredient extracted from the natural plant Artemisia annua, and this metabolic form in vivo with the most potential action is referred to as Dihydroartemisinin (DHA). A study with U87 and A172 cell lines analyzing protein expression patterns suggests that DHA activates ferroptosis through the inhibition of GPX4 in glioblastoma. Using DCFH-DA and BODIPY-C11 probes, the combination of ferrostatin-1 and DHA resulted in a reversion of the increased intracellular ROS and lipid peroxidation levels caused by DHA single treatment, corroborating the ferroptosis action in these cells after DHA treatment [99]. 

Accordingly, another study with DHA using U251 and U373 cell lines demonstrated that this drug can induce ferroptosis by causing stress in the endoplasmic reticulum (ER) in glioma cells. However, ER stress causes unfolded protein response (UPR), which could be responsible for providing drug-resistant capacity and more tumorigenicity. UPR signaling can be mediated by the PERK protein, and this pathway may mitigate the effects of DHA in glioma cell lines. Therefore, the treatment with this drug also activated, in parallel, a pathway that protects glioma cells from this cell death type: a feedback pathway of ferroptosis. This process is dependent on the PERK (protein kinase R-like ER kinase) activity, which induced HSPA5 (heat shock protein family A member 5) expression through *ATF4* activation; therefore, the PERK-ATF4 pathway resulted in the induction of *HSPA5* expression, and this led to the expression and activity of GPX4. Thus, ER stress induced by DHA causes the activation of this molecular cascade (PERK-ATF4-HSPA5-GPX4), which inhibits ferroptosis, through the prevention of lipid peroxidation. In essence, the blockage of the PERK-ATF4-HSPA5-GPX4 pathway using siRNA or small molecules could improve the DHA effect in glioma cells, increasing ferroptosis in vitro and in vivo and carrying out antitumor activity [100]. 

15,16-Dihidrotanshinone I (DHI) is another natural herbal compound (extracted from Salvia miltiorrhiza Bunge). Currently, it is used to treat cardiovascular disease and has been studied due to its therapeutic effects on some types of cancer cells. It was observed that DHI decreases the cell proliferation in a dose- and time-dependent manner in the U251 and U87 cell lines. The results indicate that cell death occurred by GPX4 inhibition and ACSL4 increase. Furthermore, the cellular antioxidant system GSH/GSSG levels and mitochondrial membrane potential (MMP) was reduced, promoting ferroptosis [54]. Pseudolaric acid B (a diterpene acid isolated from the root and trunk bark of Cortex pseudolaricis, known as PAB) can induce glioma cell death both in vitro and in vivo due to excessive H_2_O_2_ production and lipid peroxide formation, generated mainly by the iron-activated Nox4 [101]. Moreover, it was demonstrated that these effects are also generated by the depletion of GSH and cysteine through the activation of p53, which inhibits the xCT pathway [102]. 

Among the prospective drugs that can contribute to ferroptosis, ibuprofen can exert antitumor effects in many different tumor cells including glioblastoma. This anti-inflammatory drug is a potential therapeutic strategy due to its ability to induce ferroptosis through the inhibition/dysregulation of the NRF2 signaling pathway, thereby generating increased intracellular lipid peroxidation, leading to decreased viability of glioblastoma cells in vitro and in vivo. According to Gao et al., the treatment with increasing concentrations of ibuprofen decreases *NRF2*, *GPX4*, and *SLC7A11* expression in the glioma cells, resulting in ferroptosis induction [101].

As aforementioned, Kyani et al. described a novel nanomolar PDI inhibitor, 1,3,6-trimethylpyrimido[5,4-e][1,2,4]triazine-5,7-dione (35G8), which is toxic to human glioblastoma cell lines U87MG, U118MG, A172, and NU04. Interestingly, PDI is overexpressed in multiple cancer types, but particularly in glioblastoma. Thus, targeting PDI inhibitors can be a promising strategy in the treatment of the disease. The results obtained from their study pointed out that the 35G8-induced cell death in U87MG glioblastoma cells was due to autophagy and ferroptosis, since treatment with apoptosis (Z-VAD-FMK,) and necroptosis (necrostatin-1) inhibitors did not prevent cell death. 35G8 promoted the upregulation of *NRF2* response genes including two genes related to ferroptosis, *HMOX1* and *SLC7A11*, (which are known to mediate the response to oxidative stress (ROS). Protein levels of HMOX1 and SLC7A11 were also increased. Interestingly, when U87MG received treatment by DFO, the action of 35G8 was less potent than in the absence of these, indicating that 35G8 can induce ferroptosis [63].

Koike et al. suggested the application of the compound 2-nitroimidazoles in hypoxic glioma stem cells (GSCs). The group observed that 2-nitroimidazole doranidazol could induce GSC death by mitochondrial dysfunction and ferroptosis through ROS accumulation. Thus, it could be a potential target and inducer of ferroptosis in these cell types [103].

Table 1 summarizes the information about each ferroptosis-inducing compound that is described in detail above.

Certainly, it is extremely urgent to find therapeutic alternatives for the treatment of glioblastoma, considering its devasting effect on patients. Ferroptosis-inducing compounds have played an interesting role in reversing this scenario, however, it is also important to take into account the potential long-term side effects that these drugs could trigger in brain tissues. Indeed, it was identified that lipid peroxidation, glutamate abnormal levels, and elevated iron levels are common features among ferroptosis and many neurodegenerative diseases such as Parkinson’s, Huntington’s disease, motor neuron disease, and multiple sclerosis [110,111]. Notably, it was observed that Erastin treatment could sensitize neuronal cells and elevate the iron deposition in the brain as a side-effect in vivo [112]. Neuronal cells also presented vulnerability to RSL3, and GPX4 depletion caused hippocampal degeneration, lipid peroxidation, and mitochondrial damage [113]. In this sense, ferroptosis inhibitors such as Ferrostatin-1 can partially rescue these effects of GPX4 depletion [113]. Therefore, ferroptosis inducers that block GPX4 can worsen neuronal pathologies and affect brain tissues through ferroptosis.

Since there are many compounds able to potentially reverse drug resistance through ferroptosis induction in brain tumors, an intriguing question is whether these drugs could be triggering a collateral effect in normal brain tissues. Interestingly, it was demonstrated that DHA promoted a decrease in the aggregation of amyloid β plaque and neuronal loss, and therefore it is considered as a promising therapeutic drug for hypoxic-ischemic brain damage (HIBD), and Alzheimer’s disease patients [114,115]. Similarly, ibuprofen and polyphenol amentoflavone have been suggested to play potential neuroprotective effects against neurological diseases [116,117]. Considering that some ferroptosis-inducing compounds have positive as well as negative effects in normal brain tissues, the results above-mentioned demonstrate that ferroptosis has a dual effect in cancer and neurodegenerative diseases, pointing out the demand for more studies in the area.

### 5.2. Potential Biomarkers

It is known that ferroptosis could play a crucial role in several phases of the tumor [118] such as the tumorigenesis and progression [119], cell death, and drug resistance [120]. Thus, the discovery and validation of ferroptosis biomarkers are critical in order to predict tumor prognosis [90]. In this sense, studies have demonstrated the existence of metabolic changes in glioblastoma tumors, for instance, dysfunctions in cellular respiration and in the glutamine and lipid metabolism [90,121].

Recently, some studies have established ferroptosis-related genes and lncRNA signatures that may be a promising therapeutic strategy to predict the survival and prognosis among glioblastoma patients, thus improving the individual clinical outcomes (Table 2).

For example, CD44 is a transmembrane molecule known to facilitate glioma growth and proliferation due to interactions with the tumor microenvironment, and studies have revealed that it promotes the suppression of ferroptosis in cancer cells and may cause chemoresistance [129]. FADS2 (fatty acid desaturase 2) is an enzyme responsible for the desaturation of fatty acids and is upregulated in glioblastoma [130]; Yamane et al. have already described it as a key determinant of cellular sensitivity to ferroptosis in the hepatitis C virus [131]. HSPB1 (or heat shock protein 27 ‘HSP27′) activates G6PD in response to oxidative stress or DNA damage, and together, they promote the glioma development; also, *HSPB1* has another role in the activation of *G6PD*, because it contributes to the production of cellular NADPH and pentose in glioma cells [132]. These pathways have been correlated with ferroptosis, and therefore require further studies.

## 6. Conclusions and Perspectives

Glioblastomas are a severe type of glioma, which have demonstrated accentuated drug resistance. In this sense, inducing cell death is a crucial strategy to reverse this scenario. Since ferroptosis has shown an important role in cancer treatment, exploiting ferroptosis modulation has been widely encouraged in glioblastoma research. In this review, we summarized the current relevant mechanisms of ferroptosis induction in glioblastoma cells obtained up to now. The main approaches for inducing ferroptotic cell death in this type of cancer include: (1) increasing the iron levels through *DMT1, COPZ1,* and *STEAP* genes modulation, or by treatment with gallic acid and brucine; (2) elevating the lipid levels through the regulation of the key lipid genes *ACSL4* and *LOXs* as well as *CYP2E1* and *MDM2*; and (3) disrupting the lipid peroxidation repair by direct and indirect GPX4 inhibition and gene modulation by *xCT*, and *ATF4* regulation. Ferroptosis can also be modulated in gliomas by other mechanisms such as long non-coding RNAs and autophagy. All of these aforementioned pathways represent an inhibitory effect on glioblastoma, indicating new targets to be further explored.

Some new compounds capable of inducing ferroptosis in glioblastomas such as brucine, cRGD/Pt + DOX@GFNPs nanoformulation, dihydroartemisinin, pseudolaric acid B, ibuprofen, pyrimidotriazinodione, 35G8, and 2-nitroimidazoles may provide a relevant perspective for glioma treatment. Similarly, the identification of specific ferroptosis biomarkers may contribute to achieving better outcomes for glioma patients, and we indicated interesting new potential targets to glioma therapy such as *CD44*, *FADS2*, *HSPB1*, and *GSPD*, which could possibly play an crucial role in ferroptosis modulation in glioma and thus need to be additionally explored. Altogether, these findings require further clarification to better understand the specificity of the compounds, and how ferroptosis biomarkers could predict a more effective treatment to be performed in each patient individually.

## Figures and Tables

**Table 1:** The ferroptosis-inducing compounds in glioblastoma.

Compound Name	Cell Lines	Impact on Ferroptosis	FDA-Approved	Ref.
Nanodrug RPDGs (cRGD/Pt + DOX@GFNPs)	U87MG ^+^	• Depletes of GSH	N/A	[36]
Brucine	U251, U87, U118, and A172 ^+^	Causes lipid peroxidation Increases iron and H_2_O_2_ levels	YES	[37,104]
Ibuprofen	U87MG and U251MG ^+^	Decreases NRF2, GPX4, and SLC7A11 expression Increases lipid peroxidation	YES	[101]
Dihydrotanshinone I (DHI)	U251 and U87	Decreases GPX4 and GSH Increases ACSL4 Increases mitochondrial membrane potential (MMP)	N/A	[54]
Erastin	A172, U87-MG, T98G, GBM-N6 and GBM-N15	Blocks the system xCT, inhibiting cystine importation Depletes GSH, reducing GPX4 activity	N/A	[59]
Sulfasalazine	A172, U87-MG, T98G, GBM-N6 and GBM-N15	Blocks the system xCT, inhibiting cystine importation Depletes GSH, reducing GPX4 activity	YES	[59,60,62]
Sorafenib	U251, LN18, SHG-44, and rat glioma C6	Blocks the system xCT, inhibiting cystine importation Depletes GSH, reducing GPX4 activity	YES	[59,61,105]
PDI Inhibitor (35G8)	U87MG, U118MG, A172, and NU04	Upregulates *NRF2* response genes (*SLC7A11* and *HMOX1*) Increases the cellular response to ROS	N/A	[63]
Curcumin analog (ALZ003)	U87MG	Causes lipid peroxidation and augmented ROS levels Reduces the GSH/GSSG levels, regulating negatively GPX4	N/A	[98]
RAS-selective lethal 3 (RSL3)	U373, U87, U251, U87MG, and GL261 (murine glioma)	• Increases lipid peroxidation through GPX4 inhibition	N/A	[72,73]
Polyphenol amentoflavone (AF)	U251, U373 ^+^	Induces ferroptosis in an autophagy-dependent manner Increases intracellular iron levels Decreases GSH and the mitochondrial membrane potential levels	N/A	[91]
Dihydroartemisinin (DHA)	U87, U251, U373, A172, and HT22 ^+^	• Generates ROS and lipid peroxidation, inhibiting GPX4	YES	[99,100]
Pseudolaric Acid B (PAB)	Rat C6, Human SHG-44, U87, U251 ^+^	Leads to GSH and cysteine depletion, inhibiting system xCT through activation of p53 Causes accumulation of intracellular ferrous iron, activating Nox4 and leading to lipid peroxidation	YES	[102]
2-nitroimidazole doranidazol	Glioma Stem Cells (GSCs) ^+^	Decreases the mitochondrial complex activity Increases ROS and lipid peroxidation	N/A	[103]
Apatinib	U251 and U87 ^+^	Modulates the KEAP1/NRF2 signaling pathway Increases ROS and decreases GSH.	YES *	[106]
Artesunate (ART)	U251 ^+^	Modulates p38 and ERK signaling pathway Causes iron accumulation Leads to downregulation of GPX4 through the GSH depletion, thus, generating the ROS and lipid peroxidation increase	YES	[107]
Capsaicin	U251 and U87MG	Increases ACSL4 Decreases GSH and GPX4	YES	[108]
Iron oxide nanoparticles loaded with paclitaxel (IONP@PTX)	U251 and HMC3 ^+^	Increases the levels of iron ions, ROS, and lipid peroxidation Decreases GPX4 Induces the autophagy-dependent ferroptosis pathway	N/A	[109]

The columns show the compound name, the cell line submitted to the experiments, the impact of the compound on ferroptosis, and references, respectively. The “FDA approved” column refers to medicines commercially available for the treatment of several conditions, not necessarily glioma. Symbols: ^+^: tested in vivo models and able to promote similar results and/or suppress tumor volume; *: Approved by the CFDA—China Federal Drug Administration; N/A: Not available.

**Table 2:** The ferroptosis-related genes and lncRNA signature characterized as prognostic indicators for glioblastoma patients.

Number of Ferroptosis-Related Genes	Biological Markers	Database	Ref.
25	*ACACA, ACSL1, ACSL6, AKR1C3, ANO6, AURKA, BAP1, CDKN1A, CISD1, CP, CYBB, G3BP1, G6PD, GLS2, HMOX1, HSPB1, LOX, MAP3K5, PCBP1, PGD, PRNP, RB1, STEAP3, TF, TP53*	TCGA and CGGA	[97]
19	*AKR1C2, ALOX12B, ALOX5, ALOX5AP, ATP5G3, CBS, CD44, CISD1, DPP4, EMC2, FANCD2, GCLC, GCLM, HMGCR, HSPB1, LPCAT3, NCOA4, NFE2L2, SAT1*	CGGA, TCGA, GSE16011, and REMBRANDT	[90]
12	*ARNTL, CHMP5, DNAJB6, EIF2AK4, FANCD2, HSPB1, LAMP2, MAP3K5, MT3, NFE2L2, TP63, VDAC2*	FerrDb and CGGA	[122]
7	*ACSL3, CBS, CD44, FADS2, HSPB1, PGD, STEAP3*	TCGA, CGGA, and GTEx	[119]
15	*ACSL4, ATP5MC3, CISD1, DPP4, FANCD2, FDFT1, HSPA5, HSPB1, NCOA4, NFE2L2, RPL8, SAT1, SLC1A5, SLC7A11, TFRC*	TCGA and GEO database	[123]
22	*ACSL4, AIFM2, ATF4, BCL2, BECN1, FTH1, FTL, GOT1, GPX4, HSPB1, KIAA1429, NCOA4, NFE2L2, NFS1, SLC11A2, SLC1A5, SLC40A1, SLC7A11, TF ZEB1, TFRC, TP53,*	TCGA, CGGA, and ssGSEA	[118]
15	ARHGEF26-AS1, CPB2-AS1, GDNF-AS1, LINC00641, LINC00844, MIR155HG, MIR22HG, PAXIP1-AS2, PVT1, SBF2-AS1, SLC25A21-AS1, SNAI3-AS1, SNHG18, WAC-AS1, WDFY3-AS2	TCGA, CGGA, and Rembrandt	[84]
14	APCDD1L-AS1, H19, LINC00205, LINC00346, LINC00475, LINC00484, LINC00601, LINC00664, LINC00886, LUCAT1, MIR155HG, NEAT1, PVT1, SNHG18	WGCNA, CGGA, TCGA, CGGA_693, and CGGA_325	[83]
9	AC010729.2, AC062021.1, FAM225B, FAM66C, HOXAAS2, LINC00662, LINC00665, MIR497HG, TMEM72-AS1	CGGA, TCGA, and FerrDb	[124]
4	*HMOX1, JUN, SOCS1, TFRC*	TCGA, CGGA, GTEx, previously published literature, FerrDb, and ImmPort	[125]
5	*AKR1C1, AKR1C3, NCOA4, STEAP3, TFRC*	TCGA, CGGA, GEO, and previously published literature	[126]
15	*ALOX15B, ANGPTL7, CHAC1, GLUD1, lFNG, MAP1LC3A, POR, PRNP, RGS4, SLC2A1, SMPD1, STAT3, TFR2, VDR, WIPI2*	TCGA and GCGC	[127]
10	*CAPG, CD44, CDKN1A, CP, GDF15, HSPB1, LOX, MAP1LC3A, SOCS1, STEAP3*	TCGA	[128]

The columns indicate the number of ferroptosis-related genes or lncRNA signatures, the names of the genes or lncRNAs, the database analyzed, and the references, respectively. Abbreviations: TCGA: The Cancer Genome Atlas; CGGA: Chinese Glioma Genome Atlas; REMBRANDT: The Repository of Molecular Brain Neoplasia Data; FerrDb: Ferroptosis Database; GTEx: The Genotype-Tissue Expression; GEO: Gene Expression Omnibus; ssGSEA: Single-sample GSEA; WGCNA: Weighted Correlation Network Analysis; ImmPort: The Immunology Database and Analysis Portal; CGCG: Chinese Glioma Cooperative Group.
